# Engineering Judgment of Children Bone Fracture

**DOI:** 10.1155/2011/737054

**Published:** 2011-10-25

**Authors:** A. Alsamhan, M. M. ELSingergy, M. M. Zamzam, S. M. Darwish

**Affiliations:** ^1^Industrial Engineering Department, King Saud University, Riyadh 11421, Saudi Arabia; ^2^Orthopedic Surgery Department, King Saud University, Riyadh 11421, Saudi Arabia

## Abstract

Supracondylar humerus fracture (SCHF) is one of the commonest elbow fractures in children. It is common injury for children with age from four to fourteen. In current study, the finite element technique is used to evaluate two techniques, namely, parallel and crossed K-wire fixation for treatment of SCHF, using K-wire fixation.

## 1. Introduction

**Figure 1:**
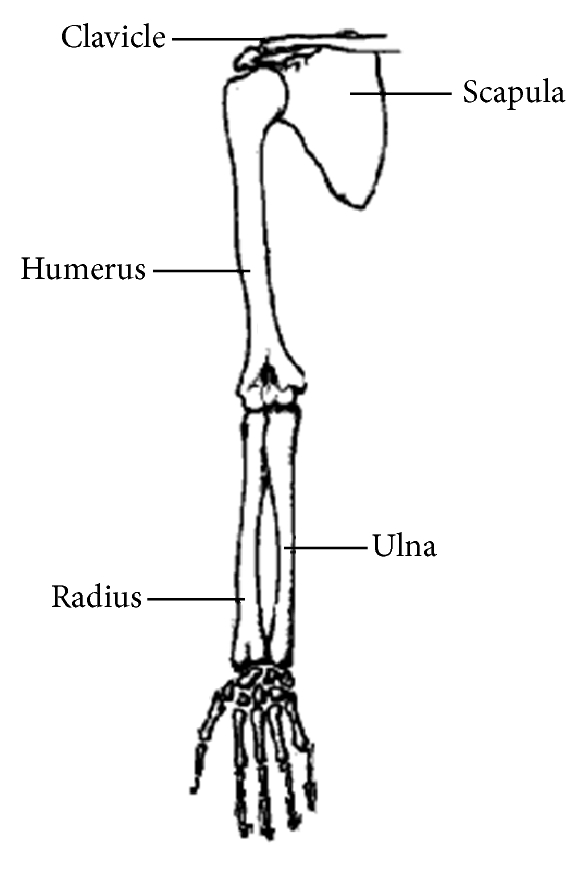
Humerus bone in human arm.

The humerus bone is the upper arm bone that connects the shoulder, by articulating the humeral head with the glenoid of the scapula, to the elbow by articulating the testal humerus with the ulna and radius, as shown in Figure 1. The supracondylar region is the area in the distal part of the humerus just above the growth plate. About 87 per cent of the elbow fractures occur in the distal humerus and about 80 per cent of them occur in the supracondylar. These fractures may be in the distal of the supracondylar region and called lower type or may be in the proximal part of the supracondylar region and called upper type [1]. 

**Figure 2:**
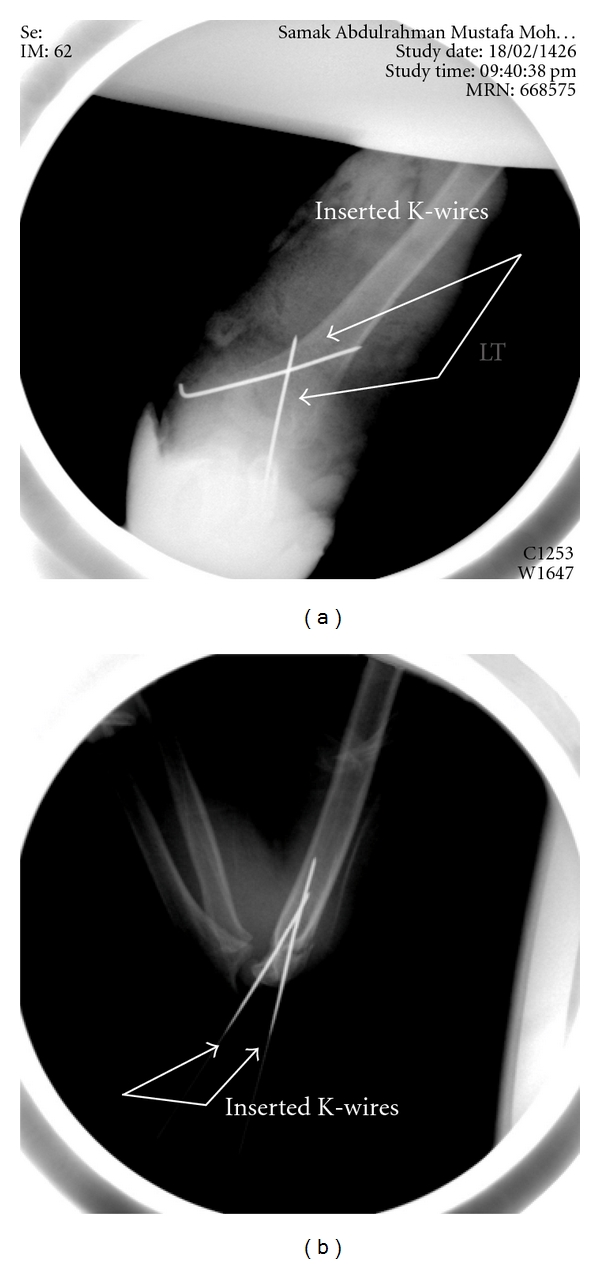
Crossing K-wires fixation treatment techniques adopted by orthopedic surgeons for distal humerus fracture treatment, (a) anterior-posterior view; (b) lateral view.

Accurate reduction and stable fixation are needed to avoid posthealing complication, namely, deformity and limitations of movements. These fractures most often require surgical treatment unless the bones are held in proper position. Sometimes surgeons want to know which alternative they should give preference, for the benefits of the patients. Fixation of unstable SCHF in children by two K-wires is the treatment of the choice by many surgeons (Figure 2, [2]).

These K-wires can be parallel from lateral side or crossing from medial and lateral sides. 

In present work, surgeon's treatment of SCHF using K-wire fixation (distal humerus type) is modeled, analyzed, and evaluated.

## 2. Research Methodology

The research role is to gather information from CT scan and X-ray developed in KKU hospital. Based on these obtained X-ray and CT scan for particular patient, a CAD solid model can be developed. The developed CAD solid model is to be coupled with the finite-element analysis to study the strength of different injury treatments adopted by orthopedic surgeons that suit that particular patient. 

The solid model and finite element models were generated using the *GID* preprocessing program [3]. The FE computation was carried out using *Tochnog* FE program [4]. *Tochnog* is explicit-implicit FE program that can be used in the analysis of structures, thermal, elastic, or elastic-plastic engineering problems. *Tochnog* and *GID *programs run under *Linux* operating system.

In the beginning, a data file of the FE model was generated using *GID* preprocessing program then completed using a text editor. Next, the *Tochnog* FE module called to run the FE analysis using the developed data file, followed by visualizing the FE results using GID postprocessing program based on the output files written by the Tochnog FE module. 

The X-ray and CT scan were obtained from KKU Hospital for humerus bone. The X-ray was obtained for two perpendicular projections. The X-ray image is imported as rusted image to AutoCAD program to measure the outer dimensions and to obtain the 2D geometry of CT scan at different levels. Next, the 2D drawing is imported to GID program and joined to complete the 3D solid model. 

**Figure 3:**
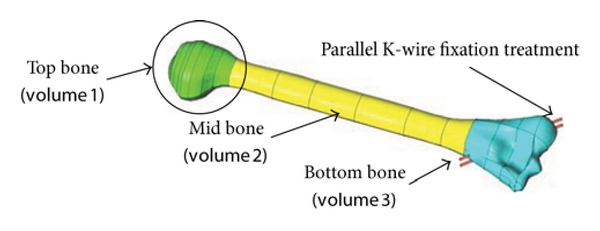
Solid model of humerus bone fracture treatment with parallel K-wires.

**Figure 4:**
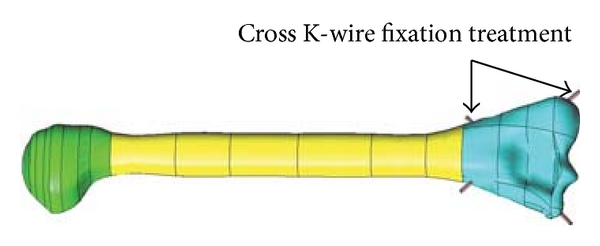
Solid model of humerus bone fracture treatment with crossed K-wires.

The solid model mainly consists of three main parts or three volumes, see Figures 3 and 4: top bone (volume 1), mid bone (volume 2), and bottom bone (volume 3).  Figure 3 shows the solid model development for the parallel K-wire fixation treatment, while, crossing K-wire fixation solid model is shown in Figure 4. 

**Figure 5:**
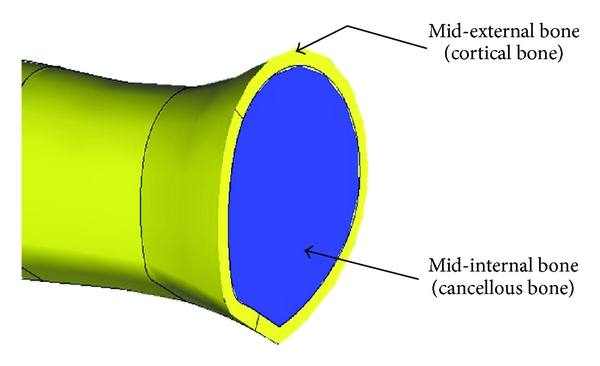
Main parts of mid-solid model of humerus bone.

The mid-part of the solid model is divided into two main parts: the external (cortical bone) part with wall thickness of 2 mm which is the harder and high strength bone, and internal (Cancellous bone) which has lower strength and hardness compared to cortical bone, see Figure 5.

## 3. Assign Boundary and Loading Conditions on the Finite Element Model

**Figure 6:**
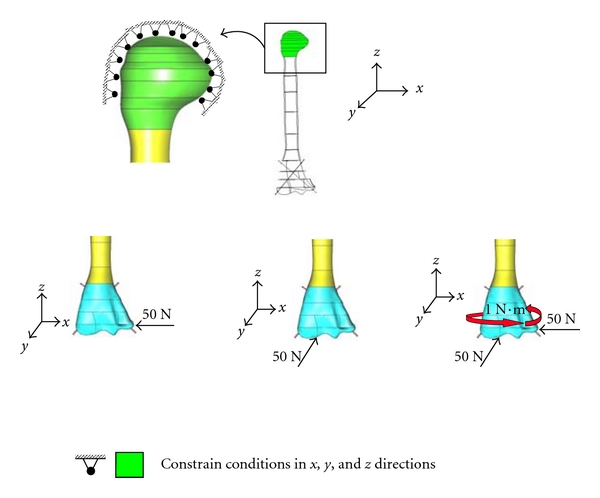
Assigned constrain and loading conditions on the developed solid models.

Constrain conditions along three directions are assigned on the top surfaces of humerus bone as shown in Figure 6. Three types of loading conditions were considered, normal load of 50 N along lateral *x*-direction, normal load of 50 N along later *y*-direction, and combined normal loads plus 1 N·M torsion load. Assigned material properties for the developed solid model are given in Table 1.

## 4. Finite Element Mesh Generation

**Figure 7:**
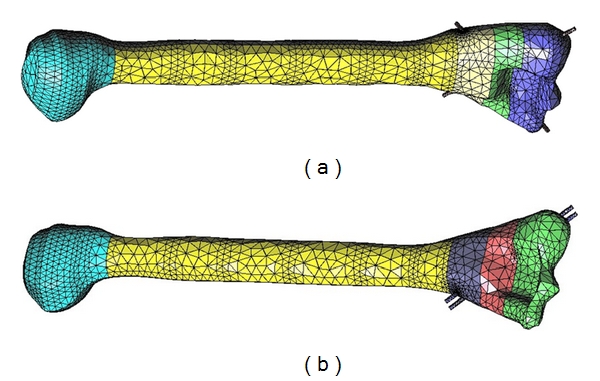
Generated finite element meshes for parallel and crossed K-wire fixation treatment models.

Two main finite element meshes were generated for modeling parallel K-wire fixation treatment model and the other for crossing K-wire fixation model. The finite element (FE) meshes were generated using 4-node tetrahedral elements, see Figure 7. The number of nodes and elements for the two FE meshes are shown in Table 2.

**Figure 8:**
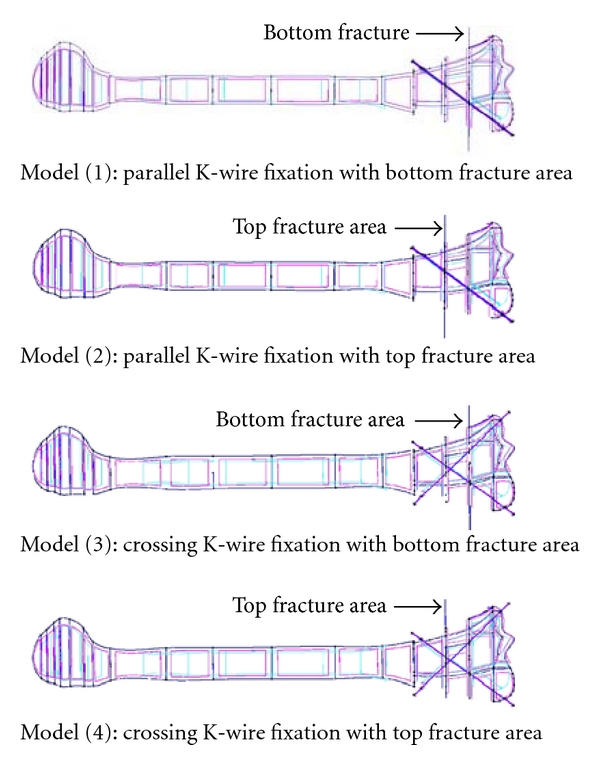
Four wire frame models to model the two types fracture areas.

Furthermore, as shown in Figure 8, four finite element models were developed to model two types of fractures at the top and bottom positions. Moreover, a friction contact surfaces were modeled in the assigned crack areas to simulate the node slipping conditions under different loads. A low friction factor of 0.1 was assigned in the crack contact areas to simulate the patient condition immediately after the surgery treatment.

## 5. Finite Element Results

**Figure 9:**
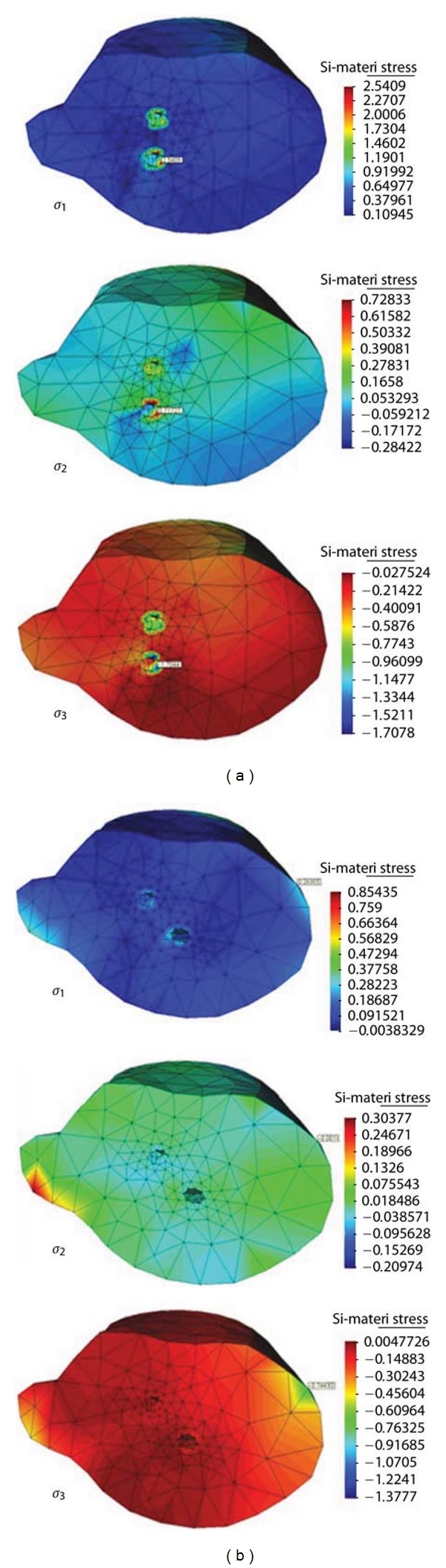
Predicted normal principal stress contours *σ*_1_, *σ*_2_, and *σ*_3_ for top fracture for normal load of 50 N along lateral *x*-direction, (a) parallel K-wire fixation, and (b) crossing K-wire fixation.

**Figure 10:**
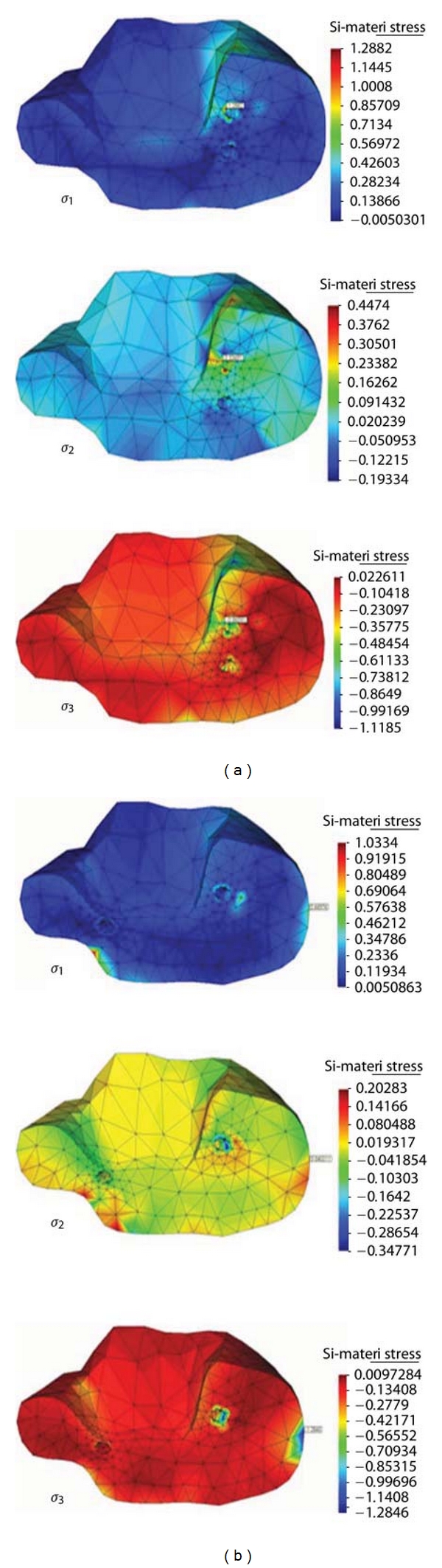
Predicted normal principal stress contours **σ**_1_, **σ**_2_, and **σ**_3_ for bottom fracture for normal load of 50 N along lateral *x*-direction, (a) parallel K-wire fixation. (b) crossing K-wire fixation.

Both peak normal principal stresses and Von-Misses stresses are reported at the two selected crack areas, see Figures 9 and 10.

**Figure 11:**
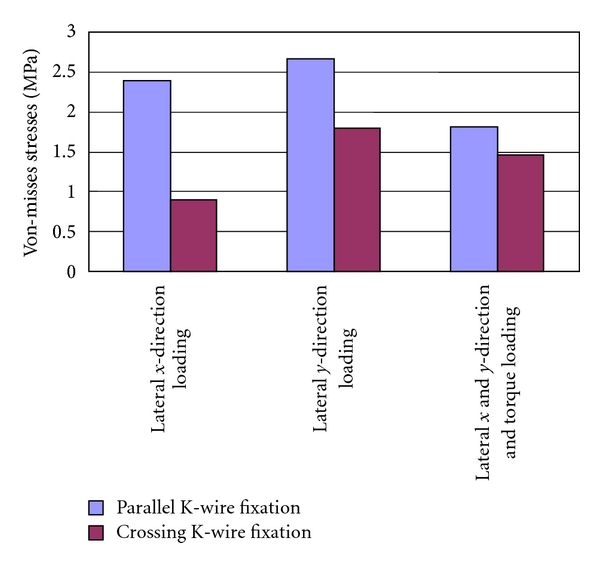
Predicted peak Von-Misses stresses for the three loading conditions for parallel and crossing K-wire fixation models, at top crack position.

**Figure 12:**
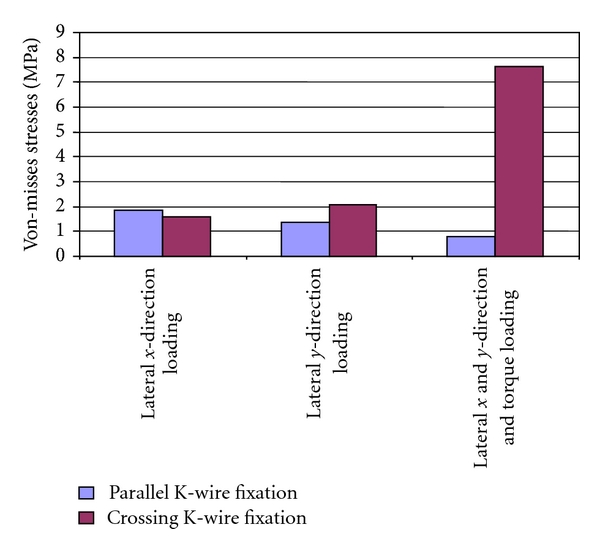
Predicted peak Von-Misses stresses for the three loading conditions of parallel and crossing K-wire fixation techniques at the bottom crack position.

Summary of the FE results for top and bottom crack positions are shown in Tables 3 and 4, respectively. Same results are also shown in charts Figures 11 and 12, respectively. The reported FE results are given for three assigned loading conditions and for two parallel and crossing K-wire fixation techniques.

Figure 11 shows the Von-Misses stresses results for top fracture area. It is clearly observed that maximum stresses on the top fracture are minimum for crossing K-wire fixation when compared with parallel K-wire fixation. These results are reported for all the three loading conditions. For example, the maximum stresses were reduced by 62% for lateral *x*-direction loading, and by 21% for lateral *y*-direction loading, and 20% for combined loading.

On the other hand, Figure 12 shows the Von-Misses stresses results for bottom fracture area. It is clearly observed that maximum stresses on the bottom fracture are minimum for parallel K-wire fixation treatment when compared with crossing K-wire fixation. For example, the maximum stresses were increased by 19% for lateral *x*-direction loading but clearly decreased by 33% for lateral *y*-direction loading and 90% for combined loading.

## 6. Conclusions

There are two types of treatment techniques commonly used for supracondylar humerus fracture in children. These treatments cover inserting crossing two K-wires or parallel key-wires fixation techniques. The present work showed that crossing K-wire fixation technique is beneficial for top fracture position, while parallel K-wire fixation technique is beneficial for bottom fracture position.

## Figures and Tables

**Table 1:** Assigned material properties on the developed solid model.

Group	Part	Material	Young modulus (MPa)	Poisson's ratio
1	Top bone	Cortical bone	3.0*E*4	0.29
2	Bottom bone	Cortical bone	3.0*E*4	0.29
3	Mid-external bone	Cortical bone	3.0*E*4	0.29
4	Mid-internal bone	Cancellous bone	5.2*E*2	0.29
5	K-wire 4 mm diameter	Nickel-chrome alloy	2.0*E*5	0.29

**Table 2:** Number of nodes and elements used to generate the two finite element meshes.

Model	Number of nodes	Number of elements
Parallel K-wire fixation treatment model	4907	21387
Crossing K-wire fixation treatment model	5660	25044

**Table 3:** Summary of FE results for top crack area.

	FE Results for different loading conditions					
*σ* (MPa), principal and Von-Misses	Lateral *x*-direction loading		Lateral *y*-direction loading		Lateral *x* and *y*-direction and torque loading	
	Parallel K-wire fixation	Crossing K-wire fixation	Parallel K-wire fixation	Crossing K-wire fixation	Parallel K-wire fixation	Crossing K-wire fixation
*σ*_1_	2.54	0.26	1.87	1.21	2.04	1.44
*σ*_2_	0.71	−0.001	−0.72	−0.46	0.41	0.06
*σ*_3_	−0.17	−0.74	0.87	−0.67	0.1	−0.09
$\overset{̅}{\sigma }$	**2.39**	**0.90**	**2.25**	**1.79**	**1.80**	**1.46**

**Table 4:** Summery of FE results for bottom crack area.

	Results for different loading conditions					
*σ* (MPa), principal and Von-Misses	Lateral *x*-direction loading		Lateral *y*-direction loading		Lateral *x* and *y*-direction and torque loading	
	Parallel K-wire fixation	Crossing K-wire fixation	Parallel K-wire fixation	Crossing K-wire fixation	Parallel K-wire fixation	Crossing K-wire fixation
**σ**_1_	1.28	0.44	0.81	1.95	0.03	2.68
**σ**_2_	0.33	0.04	−0.03	0.30	−0.25	2.12
**σ**_3_	−0.86	−1.28	−0.77	−0.35	−0.87	−5.23
$\overset{̅}{\sigma }$	**1.86**	**1.57**	**1.37**	**2.06**	**0.80**	**7.65**

## References

[1] Skaggs DL, Mirzayan R (1999). The posterior fat pad sign in association with occult fracture of the elbow in children. *The Journal of Bone and Joint Surgery*.

[2] James RK, James HB, Rockwood CA, Beaty JH, Kasser JR (2001). Supracondylar fractures of distal humerus. *Fractures in Children*.

[3] *GID* ver. 6.0 software copy writes of International Center for Numerical Methods in Engineering, (CIMNE)-Edificio C-1.

[4] Roddeman D, Buyukisik O *Tochnog* FE program. http://www.tochnog.sourceforge.net/.

